# Account of a Peculiar Sort of Tumour Observed on the Heads of New-Born Infants

**Published:** 1800-11

**Authors:** 

**Affiliations:** Harburg


### *Account of a peculiar Sort of Tumour observed on the Heads of new-born Infants; by* Dr. Michaelis, of Harburg.

[ Extracted from Loder's Journal of Surgery, &c. Vol. II. No. IV. p. 657, in German. ]

THIS tumour is to be diſtinguiſhed from ſwellings on the head, with which children are ſometimes born, or that appear after a ſlow and difficult birth, occaſioned by long preſſure or the rupture of ſome blood veſſels; and it differs, by the following marks, from any other ſwelling of the head, hernia cerebri, or hydrocephalus internus.

1. By being often obſerved after very eaſy labours.

2. By not always appearing on prominent parts of the head; though this may ſometimes be the caſe, on account of their generally being remarked at the temples.

3. By commonly appearing one day after birth.

4. By being more elevated and circumſcribed than any other ſwelling on the head, and by ſhewing a perceptible fluctuation.

5. By the ſkin, with which it is covered, keeping its natural colour and ſtate, and by its being eaſily moveable on the tumour, without changing the ſituation of it, a circumſtance that ſeems to prove its being deep; the ſkin, therefore, appears to move on the tumour when the child is crying.

6. By not diſappearing or diminiſhing on the application of preſſure, and where, alſo, no ſtupor is occaſioned, a ſymptom that always takes place in a *hernia cerebri*, or *hydrocephalus internus.*

7. It differs from a tumour caused by the rupture of blood-veſſels, or from a lymphatic ſwelling by the ſingular change which the bone, on which it is ſituated, is obſerved to undergo. The external table of the bone is entirely wanting; the diploë uncovered, and the edge of the impreſſion that is thus occaſioned may be plainly felt. By this peculiar circumſtance, the tumour is particularly diſtinguiſhable from any other.

On opening it, a black and coagulated blood is found in it, lying immediately on the diploë. It is very probable, that the tumour originates from a diſeaſe of the bone; and that it is not occaſioned by the former, on account of their both being found in the ſame ſtate from the firſt moment.

To remove this tumour by diſcutients, is an attempt that has, in moſt caſes, proved ineffectual. The diſeaſe of the bone is increaſed by the preſſure of the blood exciting the abſorbent veſſels to a greater action of the bone, whereby, at laſt, a hole is occaſioned, and the brain injured by its being preſſed. The only thing that can be done, is to open the tumour, and to let out the blood, in order to prevent the farther abſorption of the bone. This operation is not without danger, on account of the loſs of blood that runs from the bone as from a ſponge; but by making the inciſions ſmall, it may in a great meaſure be avoided. It is, however, very ſeldom that it ſucceeds in healing the bone, and the children generally fall a ſacrifice to this ſingular affection. It is, fortunately, rarely obſerved; but, according to Dr. Michaelis, it ſeemed to occur more frequently at Harburg than any where elſe. He relates a caſe of this diſeaſe, which we ſhall briefly ſtate: A tumour, of the ſize of an egg, appeared on the right temple of a new born infant, the following day after a labour which had been uncommonly eaſy. As it ſhowed the abovementioned characters, there could be no doubt of its being this ſpecies of tumour. He firſt tried to diſſolve it, and a cold ſolution of ſal ammoniac and ſalt petre in vinegar, and cataplaſms of the herb *Arnica*, were accordingly applied; but having continued them nearly a fortnight without the leaſt effect, the tumour was opened, and a black, thick blood diſcharged from the wound. The bleeding, which was not very conſiderable, ceaſed on the application of alcohol, in which ſcraped linen was dipt. The wound began to ſuppurate the next day, and in twelve days it was healed up; the child felt no pain whatever, and was quite well: An impreſſion, however, remained from the want of the external table of the bone. A few weeks after the child was ſeized with a general eryſipelas, of which it died. Dr. M. being curious to inform himſelf of the ſtate of the bone where the tumour had been ſituated, cut through the integuments, when when he found the bone appearing with a rough ſurface, deprived of its external table as far as the tumour had extended, and only ſeeming to be regenerated in ſome places.

Beſides theſe innate ſpecies of tumour, ſimilar ones occur on all parts of the body, and are always combined with a loſs of the bone, cloſe to which the blood lies. They contain coagulated and liquid blood; and on opening them, a dangerous hæmorrhage is ſometimes experienced. Dr. M. relates a caſe of a man who had four of theſe tumours; one on the right breaſt, of the ſize of a large egg, by which the ribs, where it had grown, were abſorbed; another in the neighbourhood of the temporal artery; a third on the maxilla ſuperior; and the fourth in the vicinity of the arteria ſpinoſa: this was diſcovered on the diſſection of the body; and it is probable, that by its preſſure on the brain, death was brought on. None of theſe tumours had the common ſigns of aneuriſms.

